# Functional classification of proteins based on projection of amino acid sequences: application for prediction of protein kinase substrates

**DOI:** 10.1186/1471-2105-11-313

**Published:** 2010-06-10

**Authors:** Boris Sobolev, Dmitry Filimonov, Alexey Lagunin, Alexey Zakharov, Olga Koborova, Alexander Kel, Vladimir Poroikov

**Affiliations:** 1 Department of Bioinformatics, Institute of Biomedical Chemistry of the Russian Academy of Medical Sciences, 119121, Pogodinskaya str. 10, Moscow, Russia; 2 Institute of Systems Biology, Institutskaya 6, Novosibirsk, 630090, Russia

## Abstract

**Background:**

The knowledge about proteins with specific interaction capacity to the protein partners is very important for the modeling of cell signaling networks. However, the experimentally-derived data are sufficiently not complete for the reconstruction of signaling pathways. This problem can be solved by the network enrichment with predicted protein interactions. The previously published *in silico *method PAAS was applied for prediction of interactions between protein kinases and their substrates.

**Results:**

We used the method for recognition of the protein classes defined by the interaction with the same protein partners. 1021 protein kinase substrates classified by 45 kinases were extracted from the Phospho.ELM database and used as a training set. The reasonable accuracy of prediction calculated by leave-one-out cross validation procedure was observed in the majority of kinase-specificity classes. The random multiple splitting of the studied set onto the test and training set had also led to satisfactory results. The kinase substrate specificity for 186 proteins extracted from TRANSPATH^® ^database was predicted by PAAS method. Several kinase-substrate interactions described in this database were correctly predicted. Using the previously developed ExPlain™ system for the reconstruction of signal transduction pathways, we showed that addition of the newly predicted interactions enabled us to find the possible path between signal trigger, TNF-alpha, and its target genes in the cell.

**Conclusions:**

It was shown that the predictions of protein kinase substrates by PAAS were suitable for the enrichment of signaling pathway networks and identification of the novel signaling pathways. The on-line version of PAAS for prediction of protein kinase substrates is freely available at http://www.ibmc.msk.ru/PAAS/.

## Background

The reconstruction of signal transduction networks is intensively applied in different fields of biomedicine, particularly, for identification of promising drug targets. Designed for biological network analysis databases support the effective integration of huge data obtained in large-scale experiments [1,2]. However, the experimentally derived data has many gaps, which lead to difficulties in simulating the cell signaling pathways. This problem can be settled by the network enrichment with predicted interactions. In this study we propose to apply the previously published method PAAS (Projection of Amino Acid Sequences) [3,4] for the enrichment of signal transduction networks through the recognition of proteins phosphorylated by certain kinases. We applied PAAS method to TRANSPATH^® ^database to estimate its efficiency and to predict of the new interactions that could be used for the enrichment of signal transduction networks. The TRANSPATH^® ^database is manually curated information resource providing both specific and general information on signal transduction that can has also the means for network analysis [5]. TRANSPATH^® ^database is one of the most comprehensive collections of experimentally verified data on signal transduction in eukaryotic cells. Still, many signaling interactions in various cell types are not documented in TRANSPATH^®^. This gap of knowledge can hamper the analysis of signaling networks and the prediction of functionally important elements. We suppose that addition of interactions predicted by the algorithm presented here will be useful for filling up of these gaps.

Several bioinformatics approaches were applied for prediction of the new functional characteristics of proteins with the aim of determination of new network nodes and edges [6]. Using the predictive tools one can significantly enrich the database and reconstruct more relevant models. It allows detection of promising drug targets.

Several well known algorithms use the network context information based on the protein location in the network [6] and on the comparison of the networks constructed for different species [7]. Frequently, such context information is very sparse. The amino acid sequences of proteins can serve as an important informational source for increasing the reliability of predicted proteins that participate in signal transduction.

The signaling network can be represented as a series of protein-protein interactions; therefore, the methods for prediction of the interacting protein pairs can also be used for the network enrichment. Some methods are based on the calculation of co-variation of positional substitutions in aligned sequences of interacting protein families [8]. In other methods, the members of the query pair are compared to the training set with the known protein interactions [9]. PIPE-like methods [10] calculate the similarity of short regions for the input sequence pair and the training sets and estimate the putative interactions based on the resulting matrix with the number of matches above the given threshold included. PPI-SP method is also based on the sequence comparison, but each input sequence pair is represented as vector of similarity scores calculated by the Smith-Waterman alignment [11]. The prediction of interacting pairs is performed by SVM algorithm.

In the sequence-based method for prediction of protein-protein interactions the both members of each pair are compared with the sets of sequences of known interacting proteins. We used an original sequence-based method of protein classification PAAS [3,4]. In this study the training set consisted of the known protein kinase substrates, classified according to the kinase types that can be considered as recognition of substrate specificity class using only the substrate sequences. PAAS method [3,4] is particularly appropriate for the situation when the single kinase phosphorylates many different substrates and, therefore, participates in many pathways. So, the suggested method can be applied in wide area of signal transduction pathways.

Generally, the proposed positional score is close to the measures used in other approaches - summation of weights of coincided positions (e.g. BLOSUM or PAM matrices) over the sliding window. All such methods require the shifting of sequences to each other. The more sophisticated local alignment procedure can also be considered as merging the local un-gapped similarities. Unlike other algorithms, in our approach the projection scores are assigned to each position of the query sequence. The maximal value of scores is calculated for all regions containing this position. It resembles the local alignment algorithm with more simple realization. The training sequences are projected onto the query sequence, and the summarized values obtained for all positions and all training set classes are the input to the classifier. This simple procedure does not require the large memory space. Unlike the methods based on the algorithmic alignment, PAAS algorithm does not contain the time-consuming steps.

It was shown that PAAS provides high accuracy of the functional class prediction composed of homologous amino acid sequences revealing the global sequence similarity. The proteins interacting with the same protein partner can also be characterized by the global sequence similarity. However, in many cases the proteins reveal only the local similarity. We consider that the proposed approach can be useful for determination of the proteins in the interaction network.

The proposed approach was applied for prediction of new interactions in protein phosphorylation networks. The interaction cascades between protein kinases and their substrates play a key role in cell cycle regulation, in the normal and tumor cells [12]. Protein phosphorylation (including substrate specificity of different protein kinase types, phosphorylated peptides and regions responsible for kinase-substrate binding) is well studied, providing a lot of information necessary for the evaluation and improvement of the method. The proteins included into the training set were classified according to the kinase's specificity, so that each class consisted of the proteins phosphorylated by the same kinase.

The common approach for prediction of protein kinase substrates involves the recognition of specific regions in amino acid sequences. The data set of experimentally determined phosphorylated peptides is used to compose the sequence motifs surrounding the modified Thr, Ser or Tyr residues. However, the phosphorylation motifs are not sufficient for provision of strongly specific interaction of the kinase and its substrates. The additional regions located in the substrate proteins are responsible for the enzyme recruitment, i.e. for increasing the probability of binding between kinase and substrate [13].

The algorithms based on the recognition of phosphorylation motifs and other interaction regions are used for searching of these motifs in the annotated sequences. The software like ScanSite [14], NetPhosK [15], PredPhospho [16] use the different mathematical approaches including Hidden Markov Models or Support Vector Machine. They provide the prediction of the substrates of certain kinases with high accuracy on the basis of sequence mapping [17]. In contrast to the above mentioned methods the data from the signal transduction networks frequently do not allow to make the sequence mapping. In this study we investigated the efficiency of our approach, if the amino acid sequences of training set were not mapped.

At the first stage of this study, we validated PAAS method on the basis of the known kinase-substrate interactions. At the second stage, we applied the suggested approach for prediction of new interactions for the proteins stored in TRANSPATH^® ^database. At the third stage, the predicted interactions were used for the enrichment of network. It helped us to reconstruct potential cell signaling cascades.

## Methods

### Sequence local similarity score

**Figure 1:**
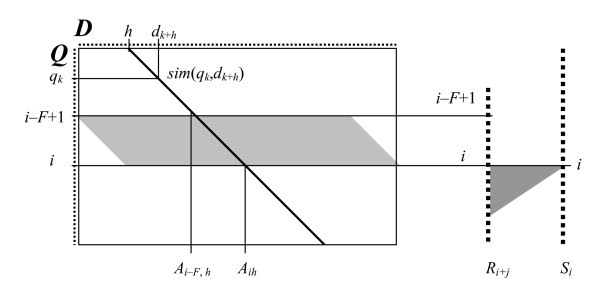
**Local similarity estimation**. The diagonal corresponds to the shift value *h *providing the best match between the region of sequence *Q *and sequence *D*. *A*_*mn *_is the summarized similarity of superposed areas of sequences *Q *and *D *terminated at *q*_*m *_and *d*_*n+h*_, respectively. Thus, the score *R*_*i *_= *A*_*ih *_- *A*_*i-F, h*_, presents the highest similarity score being found for the selected region of sequence *Q*. Finally, the similarity score S_*i *_takes the maximal values from *R*_*i+j *_scores.

In PAAS algorithm, the query amino acid sequence is described by the series of local similarity scores [3]. These values are defined by shifting the sequence *D *(retrieved from the training dataset) versus the query sequence *Q *(Figure 1). The score of similarity with the sequence *D *is calculated for each position *i *of sequence *Q *as follows:

where *sim(q, d) *is the similarity of superposed amino acid residues according to the given measure - e.g. the residue identity or substitution matrix; *q*_*x *_and *d*_*y *_are the residues in the indexed positions of *Q *and *D*, respectively; *h *is the current shift value; *F *is the value given by the parameter "frame"; *R*_*i *_is the score of maximal similarity of the sequence *Q *region (equal *F *in length and terminated at position *i *upright) with sequence *D*; *S*_*i *_is defined as maximal value of scores *R*_*i+j *_calculated for all regions, which include the position *i*.

In this study, all sequence comparisons were performed by residue similarity measure on the basis of Blosum62 matrix [18].

### Prediction algorithm

We used the algorithm described in detail in our previous publications [3,4]. The query sequence *Q *is compared to each sequence of the training set. Thus, we obtained the local similarity scores for the sequence *Q *with all training sequences. These values were used as the input data for the classifier. Belonging of the query protein *Q *to class *C *is estimated by special statistic *B_Q_(C) *[3,19-22] calculated as follows:

where *N *is a number of amino acid sequences in the training set; *W_k_(C) *and *W_k_(¬C) *are the weights of the *k*^*th *^training sequence in class *C *and its complement (in simplest case takes the value 0 or 1), *S*_*ik *_is a similarity score in position *i *of the query sequence with the *k*^*th *^training sequence, *n *is a number of amino acid residues in the sequence *Q*.

The qualitative results of prediction ("belong or not belong") are calculated for each class of proteins. The prediction result is presented in PAAS by the list of classes with the probabilities of belonging to the particular class and its complement - *P*_1 _and *P*_0, _respectively. *P*_1 _and *P*_0 _are the functions of *B-*statistic for the query sequence. The list is arranged in descending order of *P*_1_-*P*_0_; thus, the more significant results are at the top of the list. The default cut-off is *P*_1 _> P_0_.

The relationships necessary for estimating the *P*_1 _and *P*_0 _probabilities, are determined by Leave-One-Out Cross-Validation (LOO CV) procedure as follows. One sequence is removed from the training set and is used as the query set. The *B-*statistic values are calculated for each class *C *of the training set. The procedure is repeated for each sequence of the training set. Using the calculated *B-*statistic values, smooth estimations of the distribution functions *P_1_(B) *and *P_0_(B) *are obtained for each class [19,20]. Substituting the arguments for *B_Q_(C) *we can estimate the probability of the query protein belonging to the given class. This training procedure enables to save statistical model, which can be used for the estimation of new proteins.

### Evaluation of prediction accuracy

LOO CV and multiple splitting of the initial data on the training and test sets with calculation of Invariant Accuracy of Prediction (*IAP*) criterion were used for the evaluation of prediction accuracy. IAP is calculated as the ratio between the number of correctly classified pairs and that of all possible pairs [20,22]:

Mathematically, IAP value is equal to the sample estimation of the probability when the classifier ranks of the randomly chosen member *M *for the given class *C *are higher than the randomly chosen member *U *of the class complement ¬*C*. Formally, IAP criterion coincides with the Area Under the ROC Curve (AUC), which is very popular for the accuracy evaluation [23], but calculation of the IAP criterion is more simple.

### Data on protein kinase substrates

The substrates of different protein kinase types, phosphorylating the Ser/Thr and Tyr residues were studied. Phospho.ELM database [24] was chosen as the source of information with experimentally confirmed protein substrates of the known Ser/Thr and Tyr protein kinases. We selected the substrates of 45 protein kinase types: each class of kinase-specificity contained at least 10 proteins. The list of selected proteins (as designated in Phospho.ELM is presented in Table 1.

**Table 1:** Designations and descriptions of kinases whose substrates were included into the training set

Kinase type	Description	**L**_**min**_	**L**_**max**_
ABL1	Proto-oncogene tyrosine-protein kinase	178	1271

ATM	Ataxia telangiectasia mutated	118	3056

AURORA_A	Serine/threonine-protein kinase 6 (STK6)	136	1863

AURORA_B	Serine/threonine-protein kinase 12 (AURKB)	136	923

CAM_KII_group	Calcium/calmodulin-dependent protein kinase II	52	5037

CAM_KII_alpha	Calcium/calmodulin-dependent protein kinase II alpha	52	4967

CDK1	Cell division control protein 2 homolog (Cyclin-dependent kinase 1)	107	4684

CDK2	Cell division protein kinase 2	119	1971

CDKgroup	Cyclin-dependent kinases	149	1863

CK1alpha	Casein kinase 1, alpha	140	911

CK1group	Casein kinases 1	195	2843

CK2group	Casein kinase 2	98	2346

DNA_PK	DNA-dependent protein kinase catalytic subunit	270	4128

EGFR	Epidermal growth factor receptor (Receptor tyrosine-protein kinase ErbB-1)	76	1291

ERK2	Mitogen-activated protein kinase 1	196	2225

ERK1	Mitogen-activated protein kinase 3	168	2749

FYN	Proto-oncogene tyrosine-protein kinase Fyn	164	2758

GSK3beta	Glycogen synthase kinase 3 beta	164	2470

GSKgroup	Glycogen synthase kinases 3	157	1914

INS_R	Insulin receptor	132	1382

JNK1	c-Jun N-terminal kinase 1	196	1242

JNK2	c-Jun N-terminal kinase 2	196	1075

LCK	Lymphocyte-specific protein tyrosine kinase	220	2472

LKB1	Serine/threonine kinase 11 (LKB1)	433	1263

LYN	Tyrosine-protein kinase Lyn	202	1827

MAPKAPK2	mitogen-activated protein kinase-activated protein kinase 2	168	1807

MAPKgroup	P38, JNK and ERK	136	1914

PAK1	Serine/threonine-protein kinase PAK1	89	2647

PDK-1	3-phosphoinositide dependent protein kinase 1	268	1374

PKAalpha	Protein kinase, cAMP-dependent, catalytic, alpha	52	2749

PKAgroup	cAMP-dependent protein kinase	30	5037

PKBgroup	Protein kinases B	130	5890

PKCalpha	Protein kinase C, alpha type	72	2441

PKCbeta	Protein kinase C, beta 1	149	1531

PKCdelta	Protein kinase C, delta type	187	2414

PKCgroup	Protein kinase	30	2442

PKCzeta	Protein kinase C, zeta type	147	1242

PKGgroup	cGMP-dependent protein kinases	90	5037

PLK1	Polo like kinase 1	163	3418

ROCKgroup	Rho-associated, coiled-coil containing protein kinases	309	737

RSKgroup	Ribosomal protein S6 kinases	198	2647

SGKgroup	Serum/glucocorticoid regulated kinase	341	3144

SRC	Proto-oncogene tyrosine-protein kinase Src	101	4544

SYK	Tyrosine-protein kinase SYK (Spleen tyrosine kinase).	113	1290

P38alpha	mitogen-activated protein kinase 14	168	902

L_min _and L_max _are the minimal and maximal values of the sequence length of proteins referred to the given class.

**Figure 2:**
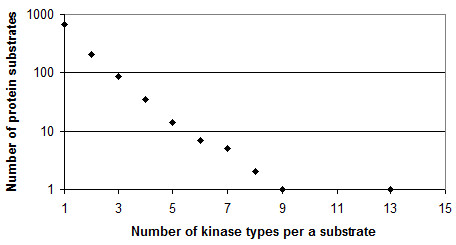
**Intersection of the kinase substrate classes**.

The UniProt accession numbers of protein substrates were retrieved from Phospho.ELM and the corresponding sequences were included into the non-redundant dataset of 1021 proteins. The obtained training set contained the proteins of the following species: the major part (971) belonged to the mammals including 709 human proteins; the remaining sequences related to other vertebrata, fungi, viruses and insects. Thus, 45 intersecting kinase specificity classes were composed (each class contained at least 10 proteins). As can be seen from Table 1, the sequence length significantly varies within each class. The average number of kinase types per one substrate protein was 1.6. The distribution of the number of kinase types per substrate is shown in Figure 2.

The certain classes were the subgroups of other classes (e.g. CDK1 and CDK2 are subclasses of CDKgroup). Sequence set of the class cannot completely cover the sets of subclasses that is typical for biological databases.

### External validation set

For further prediction, we selected 186 proteins from the commercial version of TRANSPATH^® ^database (release 2009.2) not included in the training set as a test set. It is known that the substrates of kinases are involved in various important processes, like carcinogenesis, inflammation, apoptosis, etc. Therefore, the prediction of the new interactions wherein the proteins from the test set could be involved is interesting for further investigations of the appropriate processes.

### Reconstruction of signal transduction pathways

We applied the ExPlain™ software, version 2.4.1 [25], which can be used for the iterative building of the signal transduction cascades on the basis of full network from TRANSPATH^® ^database and the shortest path algorithm. The microarray data published by Viemann et al. [26] were also used in the study.

### Microarray data

We have analyzed the microarray gene expression data on TNF-alpha stimulation of primary human endothelial cells (HUVEC) taken from GEO (GSE2639) [26]. Gene expression profiles were measured by Affymetrix^® ^GeneChip^® ^Human Genome U133A array in HUVEC, stimulated for 5 hours with TNF, and in untreated HUVEC too. Four repeated experiments were used for each condition. We applied the criteria of at least two-fold change in gene expression and *p*-value < 0.01 revealed by *t*-test. The expression of 74 genes appeared to be significantly higher after TNF-alpha treatment.

## Results

### Leave-one-out cross-validation

LOO CV procedure was performed for the set of 1021 amino acid sequences of protein kinase substrates assigned for 45 classes. The results obtained for different frame values are given in Table 2.

**Table 2:** IAP values obtained by LOO CV for the training set

Kinase type	Number of substrates	Frame values							
		
		10	15	20	25	30	35	40	45
ABL1	32	0.652	0.665	0.672	0.661	0.675	0.685	0.683	0.664

ATM	30	0.787	0.780	0.786	0.779	0.785	0.781	0.782	0.786

AURORA_A	12	0.747	0.732	0.743	0.792	0.784	0.773	0.769	0.744

AURORA_B	14	0.857	0.840	0.819	0.858	0.871	0.879	0.871	0.876

CAM_II_group	40	0.689	0.708	0.699	0.707	0.680	0.692	0.693	0.703

*CAM_KII_alpha*	21	0.616	0.592	0.591	*0.531*	*0.532*	0.528	0.519	0.529

CDK1	69	0.640	0.645	0.641	0.648	0.656	0.657	0.662	0.658

CDK2	28	0.767	0.747	0.754	0.761	0.753	0.748	0.730	0.725

CDKgroup	47	0.693	0.715	0.702	0.682	0.664	0.663	0.667	0.670

CK1alpha	11	0.578	0.553	0.575	0.609	0.625	0.642	0.594	0.560

CK1group	18	0.642	0.644	0.639	0.637	0.627	0.630	0.660	0.662

CK2group	122	0.745	0.740	0.735	0.746	0.742	0.737	0.742	0.748

*DNA_PK*	11	0.492	0.506	0.458	*0.508*	*0.529*	0.563	0.537	0.545

EGFR	27	0.840	0.843	0.883	0.861	0.893	0.887	0.891	0.888

ERK2	71	0.714	0.700	0.695	0.697	0.698	0.700	0.696	0.702

ERK1	61	0.655	0.639	0.634	0.632	0.632	0.631	0.622	0.634

FYN	25	0.687	0.697	0.695	0.696	0.651	0.627	0.619	0.632

GSK3beta	26	0.654	0.661	0.690	0.688	0.706	0.718	0.716	0.725

GSKgroup	20	0.650	0.640	0.664	0.616	0.589	0.592	0.557	0.566

INS_R	13	0.709	0.641	0.643	0.685	0.668	0.632	0.623	0.569

JNK1	20	0.762	0.773	0.754	0.766	0.783	0.770	0.777	0.779

JNK2	10	0.672	0.631	0.605	0.653	0.632	0.599	0.638	0.655

LCK	29	0.813	0.820	0.831	0.824	0.826	0.834	0.838	0.835

LKB1	16	0.996	0.995	0.994	0.994	0.993	0.989	0.990	0.991

LYN	26	0.751	0.771	0.743	0.722	0.705	0.706	0.711	0.712

MAPKAPK2	17	0.618	0.637	0.629	0.641	0.571	0.542	0.511	0.522

MAPKgroup	36	0.677	0.664	0.676	0.676	0.666	0.668	0.665	0.669

*PAK1*	21	0.500	0.517	0.570	*0.573*	*0.569*	0.575	0.576	0.541

PDK-1	24	0.957	0.958	0.956	0.957	0.955	0.948	0.949	0.950

*PKAalpha*	22	0.367	0.356	0.356	*0.409*	*0.388*	0.424	0.420	0.398

PKAgroup	206	0.658	0.660	0.669	0.668	0.672	0.648	0.646	0.648

PKBgroup	63	0.663	0.676	0.661	0.655	0.640	0.630	0.637	0.637

PKCalpha	81	0.663	0.653	0.656	0.643	0.646	0.649	0.656	0.653

*PKCbeta*	10	0.294	0.376	0.350	*0.364*	*0.415*	0.475	0.481	0.427

*PKCdelta*	17	0.418	0.472	0.449	*0.490*	*0.489*	0.463	0.479	0.493

PKCgroup	145	0.733	0.744	0.754	0.757	0.756	0.724	0.724	0.725

PKCzeta	11	0.643	0.626	0.668	0.701	0.736	0.746	0.743	0.733

*PKGgroup*	10	0.492	0.505	0.553	*0.551*	*0.594*	0.606	0.587	0.548

PLK1	18	0.678	0.628	0.670	0.718	0.731	0.721	0.688	0.704

ROCKgroup	12	0.828	0.852	0.862	0.856	0.866	0.872	0.880	0.889

RSKgroup	18	0.592	0.592	0.618	0.658	0.645	0.626	0.620	0.640

SGKgroup	11	0.738	0.749	0.699	0.695	0.699	0.699	0.699	0.683

SRC	92	0.731	0.732	0.740	0.742	0.742	0.738	0.746	0.745

SYK	21	0.741	0.729	0.752	0.766	0.775	0.764	0.744	0.750

P38alpha	24	0.726	0.720	0.723	0.737	0.741	0.728	0.705	0.717

Average		0.678	0.678	0.681	0.689	0.689	0.687	0.683	0.681

Table 2 shows that the highest average accuracy was reached at the frame equal to 25 or 30 residues. Thirty eight classes of kinase specificity were recognized with the reasonable accuracy. Seven classes (in italics) were recognized with IAP values less 0.6.

### Validation with multiple splitting

The procedure of multiple splitting of the initial data on the training and test sets (2/3 and 1/3, respectively) was applied for the estimation of the robustness of PAAS method. In this test we have used the total evaluation set of 1021 sequences, which represents the substrates of 45 kinase types. The subset of 907 human proteins was also used in the study. Twenty random divisions were made for each kinase type with the frame value = 25. The results are shown in Table 3.

**Table 3:** IAP values obtained by 20-fold multiple splitting

Kinase type	All species				Human			
	
	No	LOO CV	M	SD	No	LOO CV	M	SD
ABL1	32	0.661	0.685	0.072	24	0.600	0.600	0.106

ATM	30	0.779	0.751	0.086	29	0.771	0.762	0.075

AURORA_A	12	0.792	0.777	0.130	-			

AURORA_B	14	0.858	0.857	0.114	11	0.808	0.815	0.115

CAM_II_group	40	0.707	0.657	0.084	19	0.651	0.681	0.090

CAM_KII_alpha	21	0.531	0.515	0.120	16	0.475	0.494	0.111

CDK1	69	0.648	0.641	0.049	62	0.679	0.653	0.064

CDK2	28	0.761	0.740	0.066	21	0.659	0.666	0.092

CDKgroup	47	0.682	0.653	0.065	30	0.546	0.534	0.080

CK1alpha	11	0.609	0.595	0.134	10	0.548	0.535	0.097

CK1group	18	0.637	0.622	0.158	10	0.487	0.508	0.182

CK2group	122	0.746	0.734	0.041	87	0.680	0.670	0.046

DNA_PK	11	0.508	0.466	0.121	-			

EGFR	27	0.861	0.808	0.090	21	0.723	0.728	0.092

ERK1	71	0.697	0.624	0.048	54	0.621	0.637	0.054

ERK2	61	0.632	0.673	0.038	52	0.662	0.656	0.048

FYN	25	0.696	0.726	0.083	19	0.625	0.664	0.096

GSK3beta	26	0.688	0.671	0.087	20	0.606	0.588	0.125

GSKgroup	20	0.616	0.599	0.113	13	0.648	0.620	0.183

INS_R	13	0.685	0.583	0.133	-			

JNK1	20	0.766	0.763	0.093	15	0.786	0.759	0.121

JNK2	10	0.653	0.634	0.125	-			

LCK	29	0.824	0.795	0.058	24	0.787	0.792	0.072

LKB1	16	0.994	0.993	0.003	15	0.995	0.995	0.005

LYN	26	0.722	0.745	0.090	20	0.699	0.691	0.118

MAPKAPK2	17	0.641	0.652	0.082	15	0.590	0.596	0.081

MAPKgroup	36	0.676	0.661	0.063	31	0.625	0.614	0.089

PAK1	21	0.573	0.574	0.098	16	0.527	0.514	0.092

PDK-1	24	0.957	0.956	0.038	19	0.942	0.933	0.079

PKAalpha	22	0.409	0.466	0.108	-			

PKAgroup	206	0.668	0.655	0.028	138	0.595	0.593	0.037

PKBgroup	63	0.655	0.650	0.051	55	0.625	0.624	0.038

PKCalpha	81	0.643	0.627	0.054	68	0.648	0.630	0.053

PKCbeta	10	0.364	0.383	0.155	-			

PKCdelta	17	0.49	0.507	0.111	16	0.382	0.439	0.115

PKCgroup	145	0.757	0.755	0.036	84	0.691	0.656	0.046

PKCzeta	11	0.701	0.670	0.207	10	0.646	0.584	0.170

PKGgroup	10	0.551	0.642	0.187	-			

PLK1	18	0.718	0.661	0.163	17	0.699	0.669	0.119

ROCKgroup	12	0.856	0.839	0.155	-			

RSKgroup	18	0.658	0.644	0.095	14	0.471	0.511	0.146

SGKgroup	11	0.695	0.685	0.182	10	0.600	0.543	0.159

SRC	92	0.742	0.717	0.043	65	0.656	0.627	0.052

SYK	21	0.766	0.709	0.158	17	0.689	0.615	0.179

p38alpha	24	0.737	0.700	0.083	23	0.767	0.786	0.065

Average		0.689	0.677	0.096		0.654	0.648	0.094

The calculation was performed for the whole training set (1021 substrate proteins) and subset, including only human proteins (709). The numbers of the substrates in each class (No) are presented. IAP values were calculated by LOO CV procedure. The IAP values, averaged on 20 rounds of multiple splitting (M) and their standard deviations (SD), are presented. The kinase substrate classes containing less than 10 proteins (for human) are excluded.

Average IAP values for LOO CV and multiple splitting are sufficiently close to each other proving the robustness of the approach.

### Prediction for proteins from TRANSPATH^®^

The training set of 1021 substrates of kinases with the frame value = 25 was used for prediction of 186 proteins from the external validation set. All results, wherein *P*_1 _value exceeded *P*_0 _value, were considered as the putative substrates of kinases. 38 types of kinases from the training set with IAP value > 0.6 were selected for further investigation.

With the threshold *P*_1 _> *P*_0_, 2656 kinase-substrate interactions for 38 selected types of kinases were predicted for the test set. We found 55 phosphorylation reactions related to 30 proteins from TRANSPATH^® ^set (substrates) and to the studied kinase types. Table 4 displays 44 correctly predicted interactions mentioned in TRANSPATH^® ^annotations. Thus, the prediction accuracy for the independent external test set was 80% (44 confirmed reactions of 55).

**Table 4:** The confirmation of TRANSPATH^® ^interaction data with the PAAS prediction

Substrate Accession No in UniProt	Substrate Name in TRANSPATH^® ^database	Kinase type	***P***_**1**_-***P***_**0**_	IAP*
O15169	Axin	CK1group	-	0.637

O15169	Axin	GSK3beta	0.844	0.688

O15169	Axin	Cdk*	0.031	0.682

P24941	Cdk2	Lyn	0.525	0.722

P17302	Connexin-43	Src	0.754	0.742

P17302	Connexin-43	PKCgroup*	0.863	0.757

P17302	Connexin-43	PKCalpha	0.342	0.643

Q13158	FADD	PKCgroup*	0.446	0.757

Q13158	FADD	CK1alpha	-	0.609

P05230	FGF-1	CK2group	-	0.746

P43694	GATA-4	ERK2	0.915	0.697

P43694	GATA-4	GSK3beta	0.688	0.688

Q16665	HIF-1alpha	ERK1	0.038	0.632

Q16665	HIF-1alpha	ERK2	-	0.697

Q01344	IL-5Ralpha	Lyn	0.153	0.722

P17535	JunD	ERK2	0.749	0.697

P17535	JunD	JNK2	0.686	0.653

P17535	JunD	JNK1	0.642	0.766

Q13233	MEKK1	ABL1l	0.044	0.661

Q13233	MEKK1	PKCgroup	-	0.757

Q13233	MEKK1	GSKgroup*	0.371	0.616

O15151	Mdm4	CK1alpha	0.183	0.609

O15151	Mdm4	ATM	0.818	0.779

P27361	ERK1	Lck	-	0.824

P27361	ERK1	MAPKgroup	0.627	0.824

Q16539	p38alpha	p38aplha	0.482	0.737

Q13469	NF-AT1	JNK1	0.301	0.766

Q13469	NF-AT1	CK1group	0.664	0.637

Q13469	NF-AT1	PKCzeta	0.193	0.701

P16234	PDGFRalpha	ABL1	0.305	0.661

P09619	PDGFRbeta	ABL1	0.468	0.661

P53350	Plk1	Cdk1	-	0.648

P53350	Plk1	PKAgroup	-	0.668

P28749	p107	CDKgroup*	0.636	0.682

Q13309	Skp2	Cdk2	0.249	0.761

Q9Y6H5	Synphilin-1	GSK3Beta	0.709	0.688

Q9Y6H5	Synphilin-1	CK2group	0.429	0.746

Q93038	DR3	ERK2	0.617	0.697

P10276	RAR-alpha	MAPKgroup*	0.845	0.676

P10276	RAR-alpha	PKCgroup	-	0.757

P23771	GATA-3	MAPKgroup*	0.536	0.676

P29353	Shc-1	Src	0.881	0.742

P29353	Shc-1	ABL1	0.456	0.661

P29353	Shc-1	JNK1	0.126	0.766

P29353	Shc-1	MAPKgroup	0.028	0.676

P29353	Shc-1	Lyn	0.013	0.722

P29353	Shc-1	RSKgroup	-	0.658

P35228	NOS2	ERK1	-	0.632

Q07812	Bax	PKBgroup*	0.850	0.655

Q07812	Bax	JNK1	0.619	0.766

Q07812	Bax	MAPKgroup	0.124	0.676

Q13009	Tiam-1	PKCgroup	0.315	0.757

P05771	PKCgroup	PDK-1	0.521	0.957

P05129	PKCgamma	PDK-1	0.456	0.957

P28482	ERK2	PDK-1	0.690	0.957

*IAP values were calculated for the kinase type (specificity class) at training procedure.

The scores obtained for the correctly predicted interactions varied from 0.013 to 0.915. It should be noted that several predictions were obtained for the superclass or subclass of the kinase type, which can be determined in TRANSPATH^® ^entry (marked by asterisks).

All the interactions predicted with *P*_1 _> *P*_0 _are given in the Additional file 1: Predicted kinase substrate interactions.

### Application of predicted interactions for the reconstruction of signal cascades

Cytokines and other signal molecules bind to their receptors on the cell surface and trigger cascades of phosphorylation events inside the cell, leading to the activation or inactivation of transcription factors. Then, these specific regulatory proteins are relocated to the cell nucleus and bind to DNA sites switching on and off their target genes. Prediction of kinase-substrate interactions enriches the knowledge on potential phosphorylation cascades in cells and helps to understand the molecular mechanisms of regulation of important cellular functions in response to extracellular signals.

The set of predicted 2656 kinase-substrate interactions was used for the enrichment of network analysis of signal transduction cascades in skin cells, whose activation is triggered by the cytokine TNF-alpha. Based on microarray data [26], we have previously analyzed 74 upregulated genes (FC > 2.0) in the cell line HUVEC upon stimulation by TNF-alpha. We have also identified the transcription factor binding sites in the promoters of these up-regulated genes [27]. We have identified the most significantly overrepresented binding sites for several transcription factor's families like (NF-kappa B, STAT, AP-1, IRF, MEF2, OCT and FOX) by comparison with the promoters of the genes, whose expression has not been changed.

In order to reconstruct the TNF-alpha-triggered phosphorylation cascades leading to the activation of these transcription factors, we applied ExPlain™ to TRANSPATH^®^, before and after the enrichment by 2656 predicted kinase-substrate interactions.

**Figure 3:**
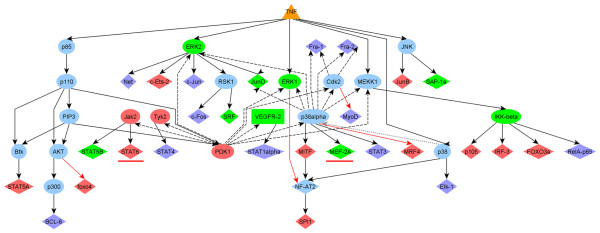
**Signal transduction cascade from TNF ligands to transcription factors reconstructed by ExPlain™ system**. TNF ligand is depicted as orange triangle. Transcription factors (TFs, diamonds) are identified by promoter analysis of up-regulated genes upon TNF-alpha stimulation of HUVEC cell line. Dashed arrows represent the novel predicted kinase-substrate interactions helping to connect TNF ligands with TFs through cascades of phosphorylation events. All other arrows represent signal transduction interactions known in TRANSPATH^®^. The up-regulated molecules are red. The down-regulated molecules are green. Two underlined TFs can be reached from TNF ligands in less than 6 steps with the help of the novel kinase-substrate interactions only.

For any set, we run twice the algorithm in downstream direction, each time starting with TNF ligand. The algorithm was stopped at reaching TF entries in the network less than 6 steps downstream off TNF. We compared two resulting networks and found that the newly predicted kinase-substrate interactions helped us to reconstruct potential signal cascades that activate several transcription factors in response to TNF, which could not be identified otherwise (Figure 3). Among such factors, we paid special attention to MEF-2A and STAT6 factors, which are known to be activated by p38alpha [28] and Jak2 [29], respectively. PAAS predicted that these two kinases can potentially be activated by PDK-1 (Figure 3, dashed arrows). Notably, with the newly predicted kinase-substrate interactions ExPlain™ reconstructed the signal cascade from TNF ligands to MEF-2A and STAT6 transcription factors identified by promoter analysis. This was not possible using the interactions documented in TRANSPATH^®^. Remarkably, there are evidences in literature on immunoprecipitation experiments showing that PDK-1 may associate with Jak2 and modulate the activity of Stat pathways [30]. The patent data have also shown that the immunoprecipitation experiments demonstrate the interaction between p38 and PDK-1 [31]. Further direct experimental studies for evaluation and validation of these predictions are necessary.

**Figure 4:**
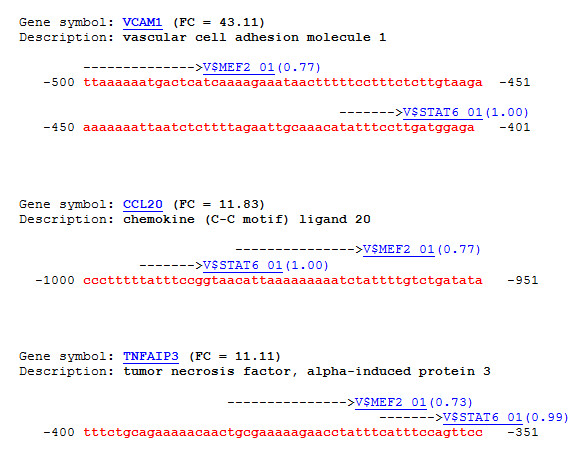
**Binding sites for MEF-2A and STAT6 transcription factors**. These binding sites are closely situated in promoters of three highly up-regulated genes upon TNF-alpha treatment. TF sites are found with ExPlain™ and position weight matrices (PWMs) from TRANSFAC^® ^database. Sites are shown as arrows above the sequences of promoters. The names of PWMs are shown together with the obtained site score (shown in the brackets).

The potential importance of MEF-2A and STAT6 transcription factors in activation of genes upon TNF treatment is demonstrated in Figure 4. We identified closely situated binding sites for these two factors in the promoters of genes characterizing extremely high fold change: VCAM1 (vascular cell adhesion molecule 1) (FC = 43.11), CCL20 (chemokine (C-C motif) ligand 20) (FC = 11.83) and TNFAIP3 (tumor necrosis factor, alpha-induced protein 3) (FC = 11.11). It is tempting to speculate that up-regulation of these genes upon TNF stimulation is triggered through the proposed here signal mechanism involving the phosphorylation of p38-alpha, Jak2 and other specific novel substrates by PDK-1 kinase.

## Discussion

The protein partner prediction is very important for the reconstruction of the cell cycle regulation network. This task is usually solved by the combination of functional characteristics and the search of specific sequence features. Significant sequence homology of the known kinase substrates and annotated protein should provide the most predictive ability. However, the large variety of proteins affected by the same kinases does not reveal the global sequence similarity.

We retrieved the kinase substrate sequences from Phosho.ELM database, as it is the most comprehensive informational resource that provides easy mining of experimentally established data. Though Phospho.ELM database contains detailed information on phosphorylated regions in the substrate sequences, we have used only the sequences classified by the kinases phosphorylating these proteins. The local similarity approach makes possible the recognition of similar regions of local sequences. We have considered that PAAS method reveals relatively short functional determinants by multiple projections of the sequences from the training set into the annotated sequence. The test with multiple divisions of the training set showed satisfactory results. When we used only human proteins removing the orthologous proteins, the results remained reasonable. So, the elimination of very similar proteins had slightly changed the kinase substrate recognitions.

The majority of existing methods for prediction of the kinase substrates is based on the recognition of the phosphorylation motifs. Corresponding sequence regions are experimentally determined. Collections of phosphorylated peptide sequences are used to construct Hidden Markov Models, Position Specific Scoring Matrices and other motif representations. Generally, the recognition properties of phosphorylation motifs are typically insufficient for the reproduction of substrate specificity [8]. The location of the kinase-docking motifs within the substrates and regulatory subunits (e.g. cyclines), substrate capturing non-catalytic interaction domain and other context information may significantly improve the prediction. The popular resource NetworKIN combines the consensus sequence motifs and protein-association networks. It increases the prediction accuracy up to 60-80% [32].

Our approach enables one to make predictions based only on the sequences of proteins, without any context data. It does not require the preliminary processing of the input data when the functional motifs should be extracted from the whole sequence. So, we showed that PAAS method can recognize protein classes consolidated by the same partners. This situation can be considered as common. Classes like LKB1, PDK-1 and EGFR substrates were recognized with very high accuracy. It can be explained by close homology of sequences in the classes. However, the classes characterized by higher variability (such as the CK2 or PKC group), were classified with the appropriate accuracy. Several kinase-specificity classes were not predicted with the appropriate accuracy (IAP < 0.6) due to the kinase substrates variability.

Prediction performed for the set retrieved from TRANSPATH^® ^database showed the possibility of our method to detect the unknown partners of certain proteins, representing a part of the known network of cell signal transduction. The results of prediction were confirmed by several TRANSPATH^® ^annotations.

Reconstruction of signal pathways may be based on the prediction of interacting protein pairs. Shen et al., using SVM-based algorithm, have accurately predicted more than 80% of interacting pairs in the three networks including 16, 189 and 93 interacting pairs. These results can be used for composition of pathways [33]. In this work, the prediction of protein-protein interactions (PPI) is based on the comparison of query pair with the training set, presenting the known interacting pairs. Such approach is used in the majority of PPI methods [9] which showed the reasonable accuracy for the large training sets [10,11]. Other authors predict the interacting proteins on the basis of interrelations of positions in the aligned sequence sets [8]. We applied the alternative approach when the proteins affected by the same kinase type are the class of kinase specificity. Thus, the prediction of kinase substrates is interpreted as classification task. It was done because significantly diverged proteins are affected by the same type of kinases presented with the small number of sequences.

In order to estimate the efficiency of our approach with regard to signaling pathways, we enhanced ExPlain™ by enriching TRANSPATH-derived data with additional PAAS-predicted interactions. The enriched interaction set was used for reconstruction of the potential signal cascades activating several transcription factors in response to TNF signaling. This approach helped us in finding the novel paths between TNF and its target genes in the cell that could not be identified otherwise. Certainly, these predictions require the experimental validation, but our study has clearly demonstrated the complementarities of approaches used by ExPlain™ and PAAS.

## Conclusions

PAAS method designed for the sequence-based recognition of functional protein classes may be used for the experimental data on the proteins participating in signal transduction. The on-line version of PAAS for prediction of protein kinase substrates is freely available at http://www.ibmc.msk.ru/PAAS/. Nevertheless the predicting results appeared to be very useful for the network enrichment and reconstruction of the signal pathways with protein-kinase substrate interactions by ExPlain™. We suggest that application of the proposed approach for the large-scale studies relative to other types of cell signal transduction should significantly help in the reconstruction of cell signaling pathways.

## Abbreviations

PAAS: Projection of Amino Acid Sequences; IAP: Invariant Accuracy of Prediction; LOO CV: Leave-One-Out Cross-Validation

## Authors' contributions

BS developed PAAS method, drafted the manuscript, collected data and performed the calculations. DF developed the classification algorithm, probability estimation of prediction and the validation method, AK applied the predicted interactions for enrichment of ExPlain™ tool, which helps to reconstruct potential signal cascades. AL conceived and designed the study; he participated in defining the format of prediction results. AZ discussed the results on each step of the study and participated in the choice of further study direction. OK performed the analysis of data to be used for prediction of new interactions and enrichment of signal transduction network. VP was responsible for overall study design and coordination. All authors read and approved the final manuscript.

## Supplementary Material

Additional file 1 **Predicted kinase substrate interactions**. File contains pairs of substrate-kinase, predicted by the PAAS. Putative substrates extracted from the TRANSPATH^® ^database are designated by UniProt Primary Accession Numbers. The kinase types are designated according to the Phospho.Elm database. The values of difference *P*_1 _- *P*_0 _are presented for prognosis estimations. So 38 kinase types recognized with IAP > 0.6 and 186 putative substrates formed the 2656 pairs predicted with threshold of *P*_1 _- *P*_0 _= 0. Click here for file
